# Durable chronic low back pain reductions up to 24 months after treatment for an accessible, 8-week, in-home behavioral skills–based virtual reality program: a randomized controlled trial

**DOI:** 10.1093/pm/pnad070

**Published:** 2023-05-23

**Authors:** Todd Maddox, Charisse Sparks, Liesl Oldstone, Roselani Maddox, Kelsey Ffrench, Heidy Garcia, Parthasarathy Krishnamurthy, David Okhotin, Laura M Garcia, Brandon J Birckhead, Josh Sackman, Ian Mackey, Robert Louis, Vafi Salmasi, Alexis Oyao, Beth D Darnall

**Affiliations:** AppliedVR, Inc., Van Nuys, CA 91406-1642, United States; AppliedVR, Inc., Van Nuys, CA 91406-1642, United States; AppliedVR, Inc., Van Nuys, CA 91406-1642, United States; AppliedVR, Inc., Van Nuys, CA 91406-1642, United States; AppliedVR, Inc., Van Nuys, CA 91406-1642, United States; AppliedVR, Inc., Van Nuys, CA 91406-1642, United States; Department of Marketing, University of Houston, C.T. Bauer College of Business, Houston, TX 77204-6021, United States; AppliedVR, Inc., Van Nuys, CA 91406-1642, United States; AppliedVR, Inc., Van Nuys, CA 91406-1642, United States; Department of Psychiatry and Behavioral Sciences, Johns Hopkins School of Medicine, Baltimore, MD 21201, United States; AppliedVR, Inc., Van Nuys, CA 91406-1642, United States; AppliedVR, Inc., Van Nuys, CA 91406-1642, United States; Division of Neurosurgery, Pickup Family Neurosciences Institute, Hoag Memorial Hospital, Newport Beach, CA 92663, United States; Department of Anesthesiology, Perioperative and Pain Medicine, Stanford University School of Medicine, Palo Alto, CA 94304, United States; AppliedVR, Inc., Van Nuys, CA 91406-1642, United States; Department of Anesthesiology, Perioperative and Pain Medicine, Stanford University School of Medicine, Palo Alto, CA 94304, United States

Dear Editor,

## Introduction

Chronic low back pain (cLBP) is prevalent worldwide.^1^ Durably effective, in-home behavioral interventions could meet the need for nonpharmacological pain care, as emphasized by the US Centers for Disease Control and Prevention (CDC) and US Centers for Medicare and Medicaid Services (CMS).^2^ Effective behavioral treatments like cognitive behavior therapy are not widely accessible, and their efficacy is inconsistent up to 12 months after treatment and rarely is examined beyond that.^3–6^ A recent randomized controlled trial in cLBP compared a US Food and Drug Administration–authorized, in-home, 8-week self-administered Skills-Based Virtual Reality (VR) program with an 8-week Sham VR program; results suggested that Skills-Based VR might address these barriers. Skills-Based VR was superior to Sham VR up to 18 months after treatment.^8–10^ The present research letter reports extended durability up to 24 months after treatment and examines the time course of “responder” rates by comparing the percentage of participants showing clinically meaningful (≥30%) reductions in pain intensity or pain interference. Previous work examining VR pain treatment durability is mixed.^7^

## Methods

Full methods have been published.^11^ The study design emerged from a collaboration between the AppliedVR Research team and Dr. Beth Darnall, Professor, Stanford University School of Medicine. Individuals with self-reported cLBP (duration of >6 months and average pain intensity of ≥4 for the past month on a 0–10 pain rating scale) were recruited through chronic pain organizations, health care professionals, and online advertisements. Once they had given their consent to participate, cLBP participants were randomized 1:1 to the Skills-Based VR Program or to Sham VR. The Skills-Based VR Program offers a fixed sequence of 56 VR experiences (to be completed once) that incorporate evidence-based self-regulatory skills used in cognitive behavior therapy, mindfulness, and pain neuroscience education. Sham VR offers a fixed sequence of 56 non-immersive, 2-dimensional nature videos overlaid with neutral music. Twenty-four–month posttreatment data from the Defense and Veterans Pain Rating Scales (DVPRS, DVPRS-II) were collected in September and October of 2022. Participants received a total of $75 for survey completion. The WCG Institutional Review Board (Puyallup, WA, United States) approved the study protocol in July 2020.

## Results

The 24-month posttreatment surveys were completed by 127 of the 168 participants who completed the end-of-treatment surveys (76%; Skills-Based VR, *n* = 68 of 84 [81%]; Sham VR, *n* = 59 of 84 [70%]). No differences in demographic or baseline clinical variables were observed between Skills-Based VR and Sham VR dropouts or between dropouts and responders within the Skills-Based VR or Sham VR groups. Demographics included age, gender, education, employment status, and income. Baseline data included pain intensity, pain interference, pain catastrophizing, pain self-efficacy, PROMIS physical function, and PROMIS sleep disturbance. Means and standard deviations are available for meta-analytic purposes.

**Figure 1.**
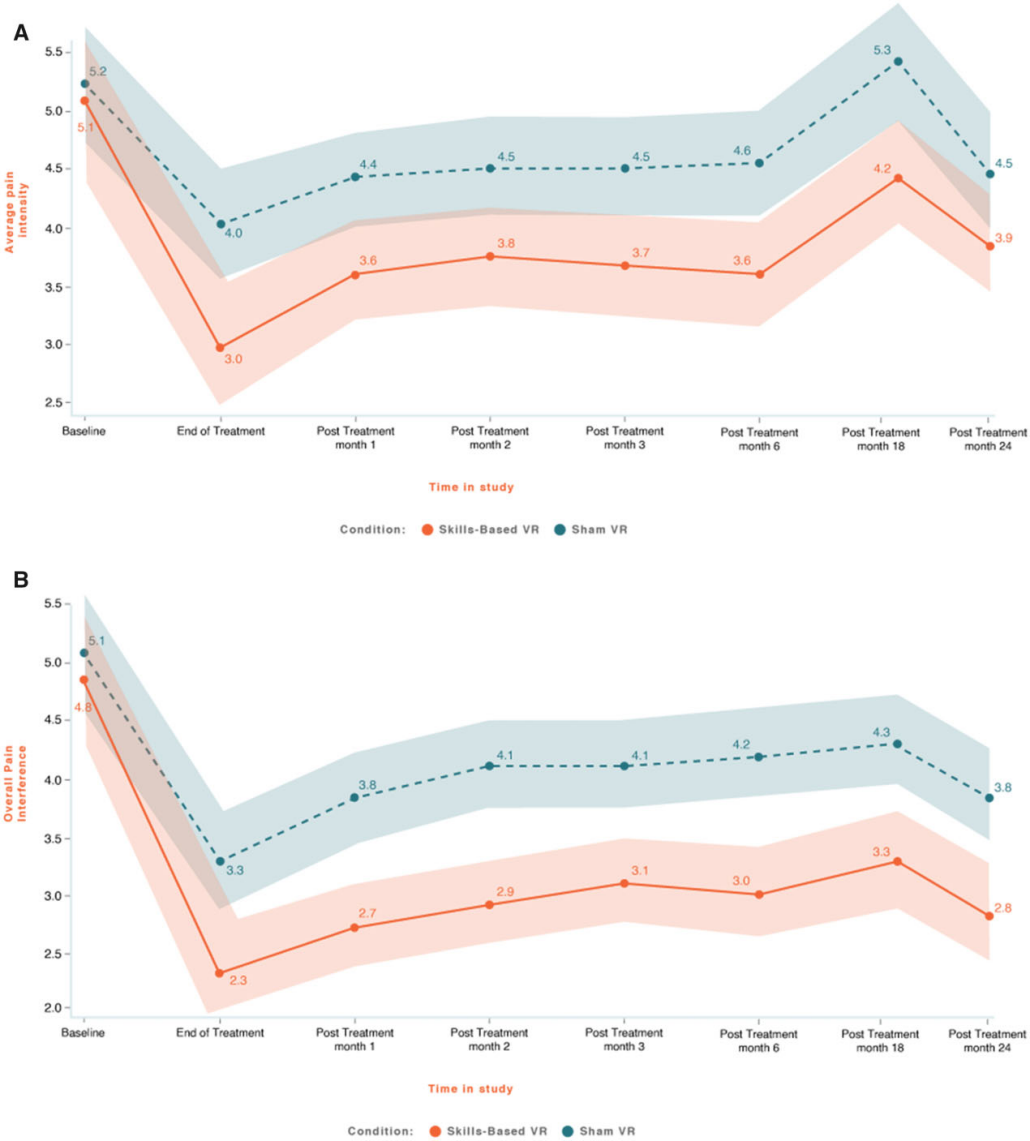
Skills-based VR and Sham VR groups from baseline to 24 months after treatment for **(A)** pain intensity and **(B)** overall pain interference. The *x*-axis represents time, and the color bands represent 95% CIs.

Figure 1 displays the group average results over time for pain intensity and the overall pain interference (average of pain interference with activity, sleep, mood, and stress).

### Skills-Based VR vs Sham VR at 24 months

Skills-Based VR pain intensity ratings (*P* = .04; Effect Size [ES] = 0.28; Skills-Based VR, SD = 1.95; Sham VR, SD = 2.05) and overall pain interference were lower than those for Sham VR (*P* = .002; ES = 0.54, Skills-Based VR, SD = 2.14; Sham VR, SD = 2.30). Pain interference subcomponents were also significantly lower for Skills-Based VR than for Sham VR: activity (*P* = .002), sleep (*P* = .011), mood (*P* = .020), and stress (*P* = .002). From baseline to 24 months after treatment, the pain intensity reduction was 24% for the Skills-Based VR group and 12% for Sham VR, and the overall pain interference reduction was 45% for the Skills-Based VR group and 21% for Sham VR. Thus, at 24 months after treatment, Skills-Based VR continued to show durable improvements in pain intensity and pain interference relative to Sham VR and relative to baseline.

### Clinically meaningful pain reductions from the end of treatment to 24 months after treatment

**Figure 2.**
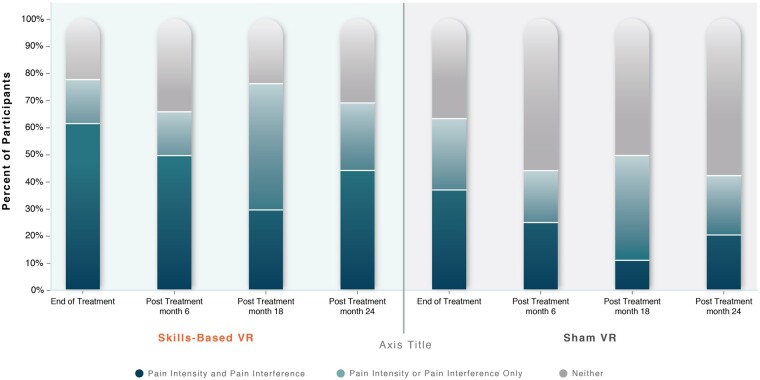
Percentage of Skills-Based VR and Sham VR participants achieving clinically meaningful (≥30%) reductions in pain intensity or (overall) pain interference from the end of treatment to 24 months after treatment.

Figure 2 displays the percentages of participants with clinically meaningful (≥30%) reductions in (1) both pain intensity and interference, (2) pain intensity or pain interference only, or (3) neither, at the end of treatment and at 6, 18, and 24 months after treatment.

Two results stand out. First, at the end of treatment, 62% of Skills-Based VR participants achieved a clinically meaningful reduction in both pain intensity and pain interference, whereas 37% of Sham VR participants did. Second, during the 6- to 24-month posttreatment period, a minimum of 65% of Skills-Based VR participants reported clinically meaningful reductions in either pain intensity or pain interference or both, whereas a maximum of 49% of Sham VR participants showed clinically meaningful reductions in pain intensity or pain interference or both.

## Discussion

The purpose of the present research letter was (1) to report 24-month posttreatment durability results and (2) to examine the time course of “clinically meaningful responder” rates from a double-blinded, placebo-controlled, and industry-sponsored randomized controlled trial comparing an 8-week self-administered, in-home Skills-Based VR with Sham VR in cLBP using the AppliedVR product. Skills-Based VR was superior to Sham VR in reducing pain intensity and pain interference at 24 months after treatment and yielded significant pain intensity and interference reductions at 24 months after treatment relative to baseline. Nearly two-thirds of Skills-Based VR participants showed clinically meaningful reductions in pain intensity and pain interference at the end of treatment, whereas just over one-third of Sham VR participants showed this pattern. Individual participant reductions remained durable, with ≥65% Skills-Based VR participants showing clinically meaningful reductions in pain intensity, pain interference, or both at each posttreatment time point to 24 months. In contrast, at no posttreatment time point did ≥50% of Sham VR participants show clinically meaningful reductions in pain intensity, pain interference, or both.

These findings suggest that Skills-Based VR might provide the durable, nonpharmacological, in-home pain relief that patients need and that the CDC and CMS recommend.^2^ We are aware of no pharmacological treatments that patients can take daily for only 2 months and gain durable pain relief 2 years later. Although one psychological nonpharmacological treatment studied in a painful health condition yielded efficacy at 24-month follow-up,^6^ high attrition (42%) was a key limitation in that study. As length of follow-up is an important measure of study quality in the Coverage and Evidence Development criteria at CMS, our 24-month data contribute substantially to our understanding of durable efficacy. Indeed, our 24% attrition rate is notably low relative to the extant literature,^3–6^ and potential attrition bias is attenuated by no between-group or within-group demographic or baseline differences for dropouts or for treatment responders. Nearly two-thirds of Skills-Based VR participants achieved a clinically meaningful reduction at 24 months. As the present study was conducted on a fairly homogeneous community-based sample, future studies should link patient-reported outcomes with health care utilization in a more broadly representative and diverse patient sample. Future work should also explore direct comparisons between Skills-Based VR and other VR pain management systems.

## Notes

**Trial registration:** ClinicalTrials.gov ID: NCT04415177; https://clinicaltrials.gov/ct2/show/NCT04415177.

International Registered Report Identifier (IRRID): RR2-10.2196/25291.

Patient enrollment began on July 1, 2020.
